# A novel *EDAR* missense mutation identified by whole‐exome sequencing with non‐syndromic tooth agenesis in a Chinese family

**DOI:** 10.1002/mgg3.1684

**Published:** 2021-05-04

**Authors:** Hongyu Zhang, Xuanting Kong, Jiabao Ren, Shuo Yuan, Chunyan Liu, Yan Hou, Ye Liu, Lingqiang Meng, Guozhong Zhang, Qingqing Du, Wenjing Shen

**Affiliations:** ^1^ Department of Prosthodontics Hebei Key Laboratory of Stomatology Hebei Clinical Research Center for Oral Diseases School and Hospital of Stomatology Hebei Medical University Shijiazhuang PR China; ^2^ Department of Orthodontics Hebei Key Laboratory of Stomatology Hebei Clinical Research Center for Oral Diseases School and Hospital of Stomatology Hebei Medical University Shijiazhuang PR China; ^3^ College of Forensic Medicine Hebei Medical University Shijiazhuang PR China

**Keywords:** *EDA* gene, *EDAR* gene, missense variant, non‐syndromic tooth agenesis

## Abstract

**Background:**

Causative variants in genes of the EDA/EDAR/NF‐κB pathway, such as *EDA* and *EDARADD*, have been widely identified in patients with non‐syndromic tooth agenesis (NSTA). However, few cases of NSTA are due to ectodysplasin‐A receptor (*EDAR*) variants. In this study, we investigated NSTA‐associated variants in Chinese families.

**Methods:**

Peripheral blood samples were collected from the family members of 24 individuals with NSTA for DNA extraction. The coding region of the *EDA* gene of the 24 probands was amplified by PCR and sequenced to investigate new variants. Whole‐exome sequencing and Sanger sequencing were then performed for probands without *EDA* variants detected by PCR.

**Results:**

A novel missense variant *EDAR* c.338G>A (p.(Cys113Tyr)) was identified in one family. In addition, three known *EDA* variants (c.865C>T, c.866G>A, and c.1013C>T) were identified in three families. Genotype–phenotype correlation analysis of *EDAR* gene mutation showed that NSTA patients were most likely to lose the maxillary lateral incisors and the maxillary central incisors were the least affected. The phenotype of mutations at codon 289 of *EDA* in NSTA affected patients was characterized by lateral incisors loss, rarely affecting the maxillary first molars.

**Conclusion:**

A novel *EDAR* missense variant c.338G>A (p.(Cys113Tyr)) was identified in a family with NSTA, extending the mutation spectrum of the *EDAR* gene. Genotype–phenotype correlation analyses of *EDAR* and *EDA* mutations could help to improve disease status prediction in NSTA families.

## Graphical abstract

A novel EDAR missense mutation, c.338G>A (Cys113Tyr), was identified in a pedigree with NSTA, extending the mutation spectrum of the EDAR gene. Genotype–phenotype correlation analyses of EDAR and EDA mutation could help to improve disease status prediction in NSTA families.

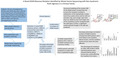

## 1 INTRODUCTION

Tooth agenesis is a pathological condition involving the absence of teeth due to a developmental failure (De Coster et al., 2009). Non‐syndromic (selective) tooth agenesis(NSTA) is one of the most common dental anomalies and is known to be associated with variants of *MSX1* (OMIM 142983), *PAX9* (OMIM 167416), *AXIN2* (OMIM 604025), *EDA* (OMIM 300451), *EDAR* (OMIM 604095), *EDARADD* (OMIM 606603), *WNT10A* (OMIM 606268) (Arte et al., 2013), *WNT10B* (OMIM 601906) (Yu et al., 2016), *LRP6* (OMIM 603507) (Ockeloen et al., 2016; Yu et al., 2019), and *GREM2* (OMIM 608832) (Kantaputra et al., 2015). Of these, *EDA*, *EDAR*, *EDARADD*, and *WNT10A* are candidate genes of both non‐syndromic tooth agenesis and syndromic tooth agenesis (STA). Ectodysplasin‐A (EDA) has been shown to bind specifically to the ectodysplasin‐A receptor (EDAR), a member of the TNF receptor superfamily, and activate the nuclear factor kappa B (NF‐κB) (Yan et al., 2000). The EDA‐EDAR‐NF‐κB signaling pathway crosstalks to the WNT and BMP pathways (Shen et al., 2016) and plays an important role in embryonic ectodermal development (Cluzeau et al., 2011; Koppinen et al., 2001).

Ectodermal dysplasia caused by *EDAR* mutations has been widely reported; according to Yu et al. (2019) about 58 *EDAR* mutations have been found in STA. However, only 12 mutations of *EDAR* have been found in patients with NSTA(Arte et al., 2013; Jonsson et al., 2018; Mumtaz et al., 2020; Zeng et al., 2017; Zhang et al., 2020). Similarly, *EDA* is the only gene known to be associated with X‐linked hypohidrotic ectodermal dysplasia, which accounts for 95% of cases of hypohidrotic ectodermal dysplasia (HED). According to Trzeciak and Koczorowski (2016), there have been 345 reported cases of HED, of which 206 are due to *EDA* mutations. As of 2017, the Human Gene Mutation Database (HGMD Professional 2017.2) had registered 314 mutations in the *EDA* gene (Reyes‐Reali et al., 2018).

In the present study, we investigate a novel missense variant *EDAR* c.338G>A as well as three previously reported missense mutations of *EDA* in Chinese Han families. The genotypes and phenotypes of all published *EDAR* mutant patients and mutations at codon 289 of *EDA* in NSTA patients were analyzed. The aim of our study is to investigate the potentially pathogenic gene mutations for NSTA, to provide a genetic mechanism and a genotype–phenotype correlation for NSTA caused by mutations.

## 2 MATERIALS AND METHODS

### 2.1 Ethical compliance

The study was conducted under the approval of the Ethics Committee of the School and Hospital of Stomatology, Hebei Medical University (NO: [2016] 004). All participants or their guardians signed written informed consent.

### 2.2 Subjects

This study involved 24 non‐consanguineous families with NSTA who were referred to the Department of Prosthodontics, School and Hospital of Stomatology of Hebei Medical University (China) from 2013 to 2019, and 100 non‐consanguineous controls. All participants were examined by prosthodontics specialists to determine the status of dentition. An oral examination and a dental treatment history were performed, and panoramic radiographs were taken to confirm the congenital absence of teeth.

### 2.3 DNA sample collection and extraction

Peripheral blood samples were obtained from all the probands as well as their parents using EDTA as an anticoagulant. DNA was extracted from leukocytes using standard proteinase‐K phenol–chloroform methods (E.Z.N.A. Blood DNA Midi Kit) and stored at −20°C.

### 2.4 PCR amplification and mutation screening

The primers used to amplify the eight coding exons of the *EDA* gene (reference sequence NM_001399.5) in PCR were based on those used by Song et al (Feng et al., 2018; Song et al., 2009). PCR reactions were elicited in a total volume of 50 μl, each containing 100 ng DNA, 4 μl of dNTPs, 5 ml 10 × TransStart Taq Buffer, 0.2 μl of each primer, and 1.25 U TransStart Taq DNA Polymerase (Thermo Fisher). After denaturing at 95°C for 5 min, amplification was carried out as follows: 35 cycles at 95°C for 30 s, 60°C for 30 s, 72°C for 30 s, and finally 72°C for 7 min. Primers of exon 4 of the *EDAR* gene (reference sequence NM_022336.4) were as follows: F: 5′‐GGCAAGAGTAGCTTCTGGAGAC‐3′; R: 5′‐GTTAATGGCCACTTAGGAGACAC‐3′. Amplification was tested by agarose gel electrophoresis and DNA was sequenced by the Beijing Genomics Institute, Beijing, China. The nucleotide sequence was analyzed using the BLAST database of the National Center for Biotechnology Information (https://blast.ncbi.nlm.nih.gov/Blast.cgi). We screened the nucleotide variants in the *EDA* and *EDAR* genes as well as 100 unrelated population‐matched controls. The sequencing results were compared with the reference sequences of *EDA* and *EDAR*, respectively. Mutation nomenclature was used, with nucleotide numbering starting with c.1 at the A nucleotide of the ATG translation initiation codon of the reference sequence.

### 2.5 Whole‐exome sequencing and Sanger sequencing

Whole‐exome sequencing was performed for 21 probands with NSTA, who did not have any *EDA* variants detected by PCR. Target enrichment and amplification were performed via liquid‐phase capture method with testing kits (iGeneTech Bioscience Co., Ltd.). The Illumina NovaSeq 6000 Genome Analyzer platform (Illumina) was used to sequence the exons from the targeted regions. With a sequencing yield of more than 17,550 Mb raw bases, the samples achieved a mean target depth of 138×. Reads were aligned to the Genome Reference Consortium Human Build 37 (GRCh37/hg19) with the Burrows–Wheeler Aligner. Single‐nucleotide variants and small indels were identified with SAMtools and Genome Analysis Toolkit (GATK) and then annotated by ANNOVAR. Candidate variants were filtered according to the following criteria: MAF < 1% and exonic. The candidate *EDAR* variant was then verified with PCR, followed by Sanger sequencing. PCR was performed and the PCR products were sequenced as described in Section 2.​4.

### 2.6 Bioinformatics analyses and structural modeling

PolyPhen 2, Sorting Intolerant from Tolerant (SIFT), and Mutation Taster were used to predict the effect of novel missense variant. A cross‐species alignment of the EDAR amino acid sequence was performed using Clustal Omega (https://www.ebi.ac.uk/Tools/msa/clustalo) with reference to the following species: human (>NP_071731.1), cattle (>XP_005212787.1), zebrafish (>NP_001108536.2), rhesus monkey (>XP_014968589.2), dog (>XP_005626028.2), mice (>NP_034230.1), and chicken (>NP_001012629.1). The structure of the ligand‐binding domain (LBD) of wild‐type EDAR was established using SWISS‐MODEL (https://swiss‐model.expasy.org). The template for modeling was based on that reported by Parveen et al. (2019). Subsequently, visualization of the three‐dimensional structure was performed with the PyMOL software (PyMOL Molecular Graphics System; DeLano Scientific).

### 2.7 Analysis of tooth agenesis patterns in NSTA patients with *EDAR* or *EDA* mutations

Genotype‐phenotype analysis was performed for 23 NSTA patients with defined *EDAR* variants; two were patients in family 4 and 21 were patients from five previous studies with detailed documentation of the tooth agenesis sites. We also collected data on six NSTA patients with *EDA* mutations at codon 289. We calculated the rate of missing teeth by counting the number of missing teeth per tooth site.

## 3 RESULTS

### 3.1 Clinical features

**FIGURE 1:**
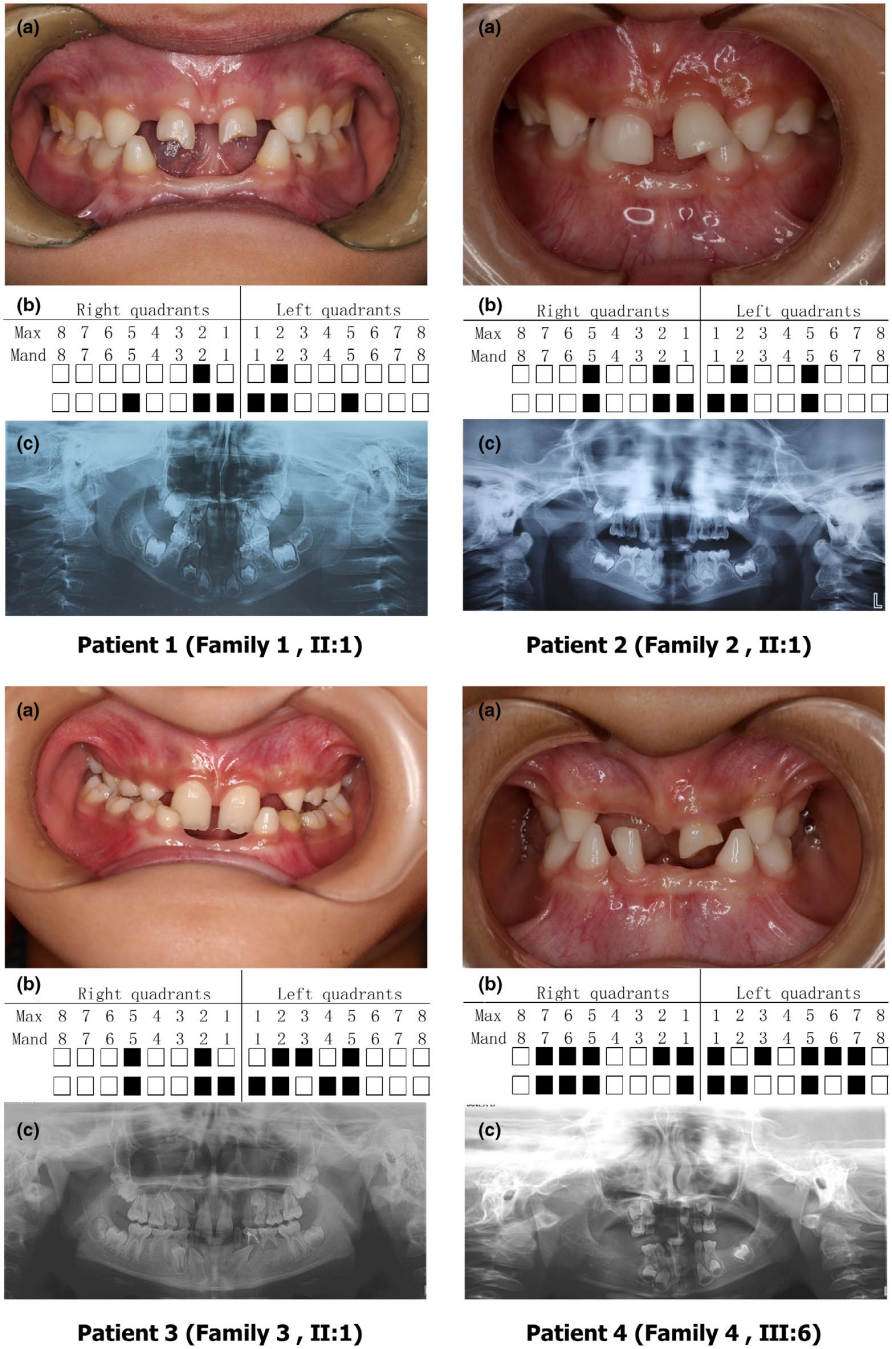
Characteristics of the four probands with non‐syndromic tooth agenesis (NSTA). (a) Intraoral image of probands; (b) Legend of intraoral missing teeth of probands; (c) Panoramic radiography of probands. Black squares indicate missing teeth; Max: maxillary; Mand: mandibular

The clinical examination of four probands with *EDA* and *EDAR* variants all exhibited congenital oligodontia, each missing 6–10 deciduous teeth and 8–18 permanent teeth (excluding the third molars, Figure 1). The loss of permanent teeth was confirmed by panoramic radiographs and found to be distributed extensively in both dentitions. However, the shapes and sizes of the residual teeth were normal. None of their parents (except the mother of proband 4) had congenitally missing teeth, and mothers did not have small or conical teeth. All participants reported normal sweating and lachrymal secretions. They had no complaints of dry mouth, intolerance to heat, or susceptibility to respiratory tract infections. The participants had hair on the body and scalp, and their facial features, skin, and nails were observed to be normal (Figure 1).

### 3.2 Mutation analysis of *EDAR* and *EDA*

**FIGURE 2:**
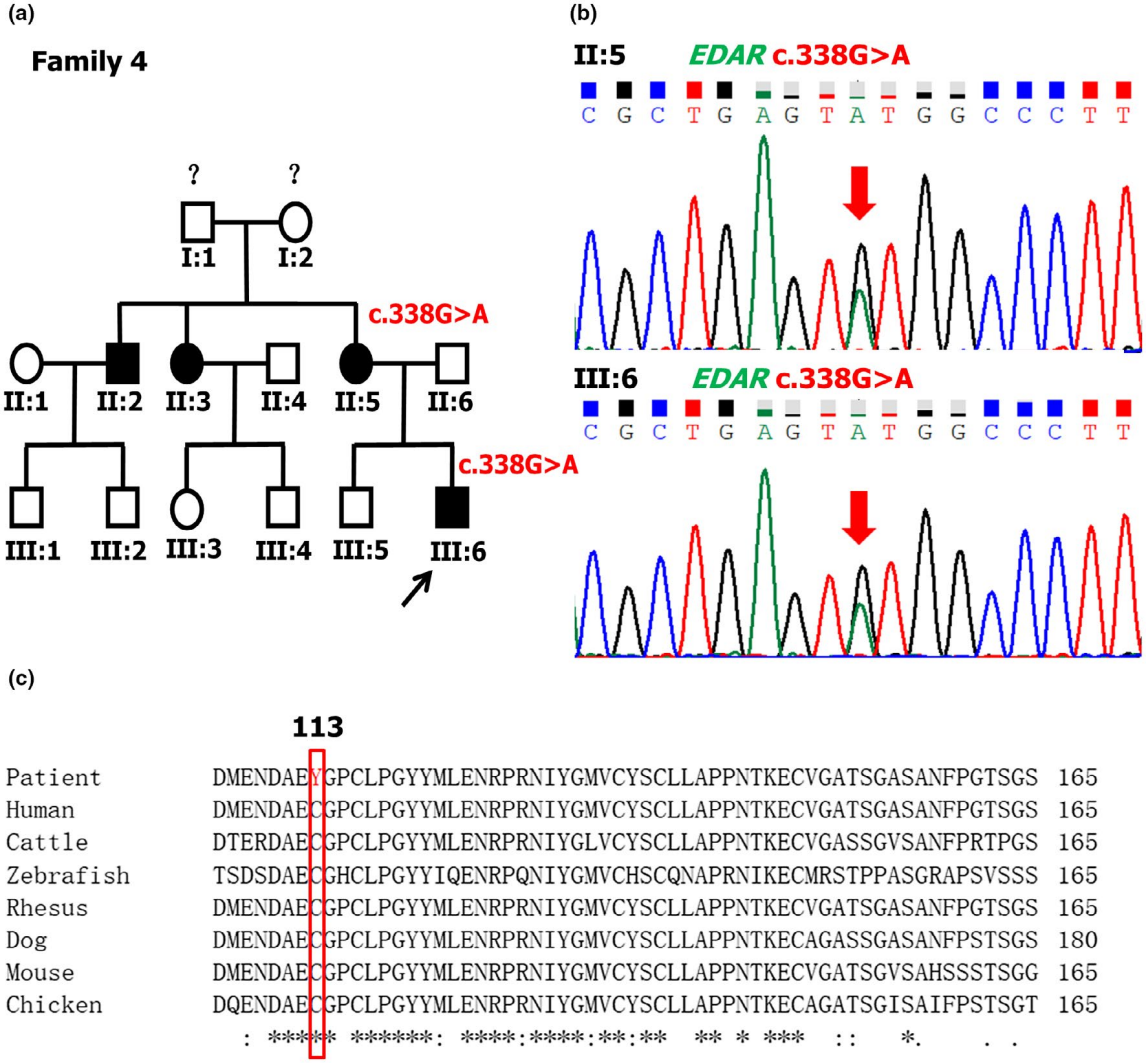
Identification of a missense variant of the *EDAR* gene in NSTA Family 4. (a) The pedigree of NSTA Family 4; the black arrow points to the proband. (b) DNA sequencing chromatograms of the proband and his mother. (c) Sequence alignment results show that p.Cys113 are conserved across seven species. The mutant allele is marked in the red box. *Indicates a completely conserved column of amino acids. The reference sequence for *EDAR* is NM_022336.4

After diagnosis, we aimed to determine the causative gene variants. After screening the coding sequences of the *EDA* gene in all probands by PCR, we found three previously reported variants of *EDA*. We then identified a novel heterozygous *EDAR* missense variant c.338G>A (p.(Cys113Tyr); NM_022336.4) (frequency <0.005 in all populations reported in the gnomAD database) in proband 4 by filtering. Furthermore, this nucleotide alteration was not found in healthy controls (n = 100) or in the NHLBI exome sequencing project Exome Variant Server (https://evs.gs.washington.edu/EVS/), indicating that the substitution was a rare variant. The candidate variant was then confirmed for proband 4 and his mother by Sanger sequencing (Figure 2b), while the wild‐type sequence was detected at this site for his unaffected father and brother. A cross‐species amino acid sequence alignment of EDAR showed that Cys113 was highly conserved among seven species (Figure 2c). SIFT, Polyphen2, and MutationTaster predicted that the mutation was “deleterious” (0.00), “probably damaging” (0.996), and “disease‐causing” (1.00), respectively, suggesting that the variant is highly pathogenic. No other candidate variants were identified in the evaluation of the exome file of proband 4 to rule out the possibility of a contribution of any variation in the other known causative genes for NSTA.

**FIGURE 3:**
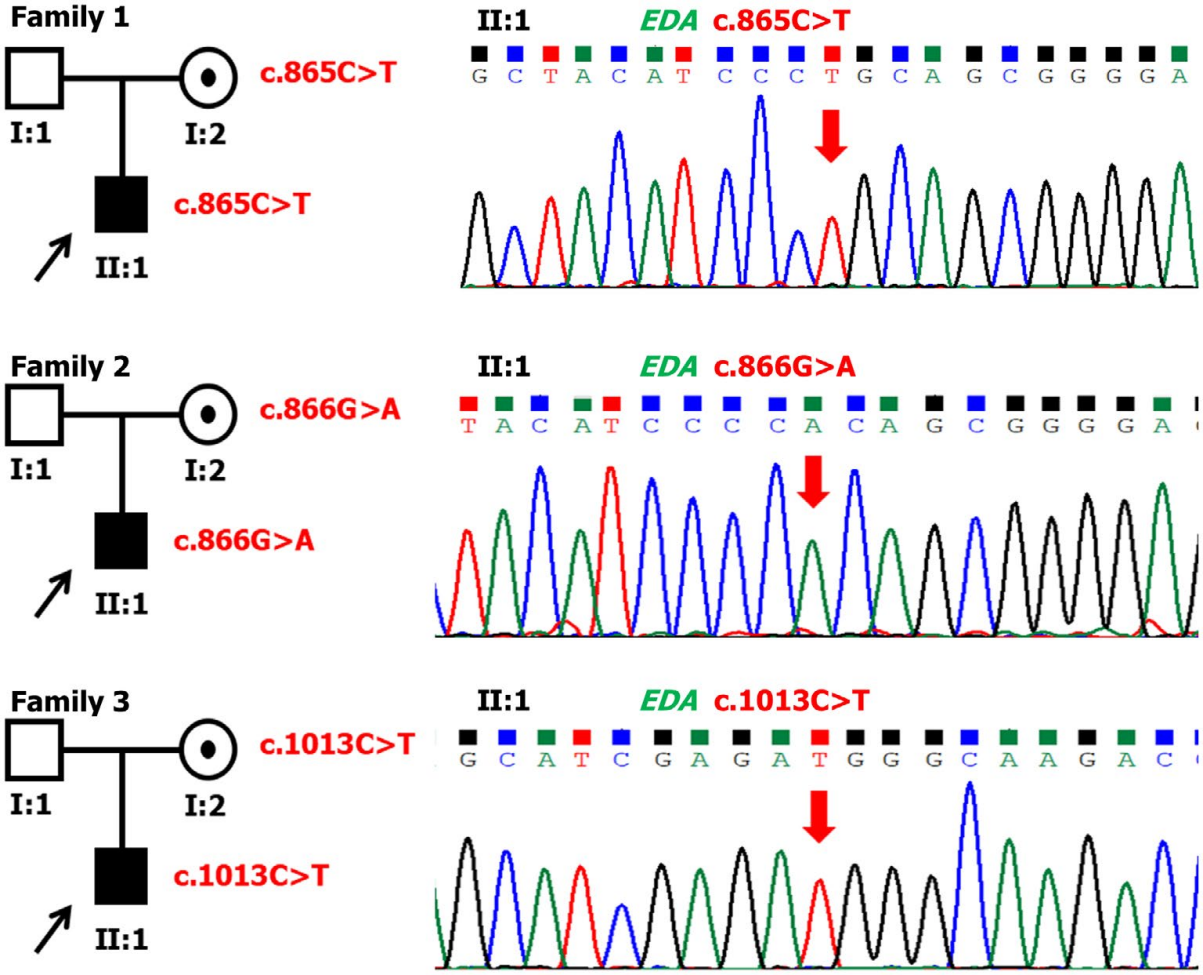
Pedigrees and variants information in NSTA Families 1–3. Black arrows point to the probands. Red arrows indicate the mutated bases in DNA sequencing chromatograms. Three reported *EDA* mutations c.865C>T (p.Arg289Cys), c.866G>A (p.Arg289His), and c.1013C>T (p.Thr338Met) were identified in the probands of NSTA families 1–3. Mothers of patients are all heterozygotes, fathers are all unaffected. The reference sequence for *EDA* is NM_001399.5

Three *EDA* mutations c.865C>T, p.Arg289Cys (Song et al., 2009); c.866G>A, p.Arg289His (Ruiz–Heiland et al., 2016); and c.1013C>T, p.Thr338Met (Han et al., 2008) (Figure 3) were found in oligodontia patients 1, 2, 3, respectively, all of which are located in the TNF domain. In Patient 1, the c.865C>T mutation occurs in exon 7 of *EDA*, changing codon 289 from encoding Arg to Cys. The c.866G>A mutation was found in exon 7 of *EDA* of Patient 2, changing codon 289 from encoding Arg to His. For Patient 3, there was a c.1013C>T mutation in exon 8 of *EDA*, changing codon 338 from encoding Thr to Met.

### 3.3 Conformational analysis of EDAR mutant

**FIGURE 4:**
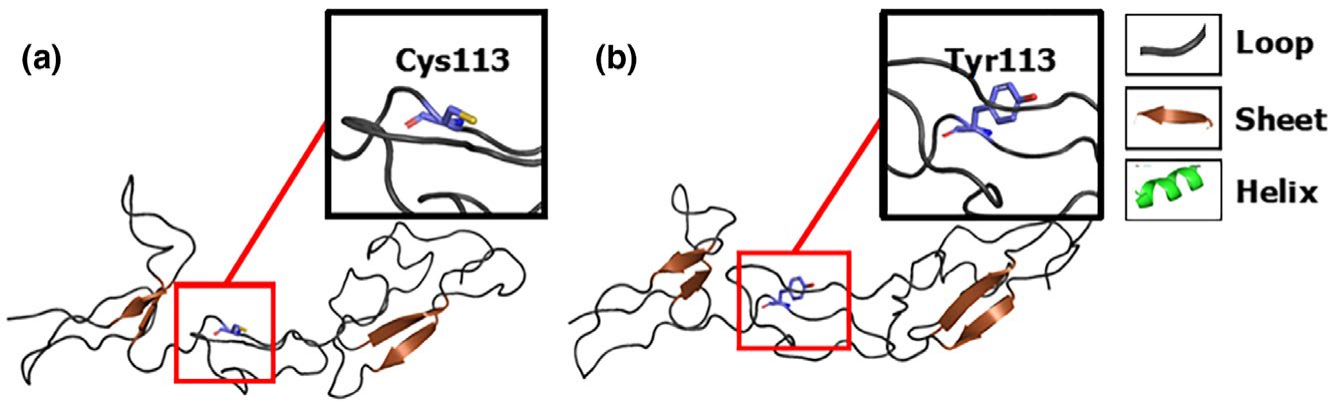
Structural modeling of the wild‐type and p.Cys113Tyr‐mutant LBD. (a) Wild‐type LBD. (b) p.Cys113Tyr‐mutant LBD. Black boxes denote the amino acid residue at codon 113 of EDAR

Structural modeling of the mutant LBD of the EDAR protein showed that the p.Cys113Tyr mutation resulted in the substitution of the hydrophobic residue Cys113 by a polar amino acid Tyr with an aromatic ring and a longer side‐chain than Cys. Based on the much larger size of the mutated p.113Tyr compared with the wild‐type p.113Cys, a significant conformational change in the LBD was predicted (Figure 4).

### 3.4 Phenotypes of NSTA patients with *EDAR* mutations

**FIGURE 5:**
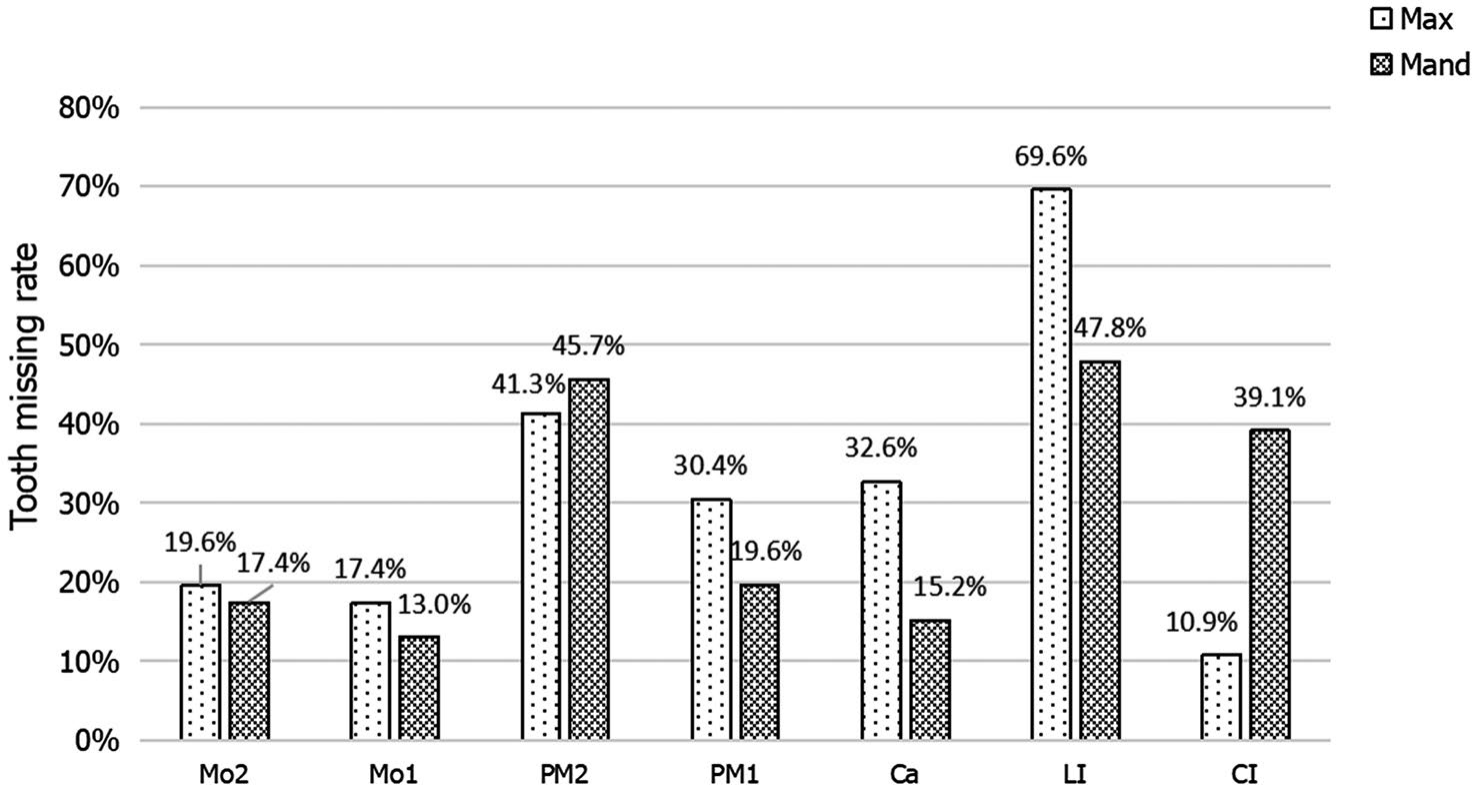
Tooth missing rate of NSTA patients with *EDAR* mutations (excluding the third molars). Ca, canine; CI, central incisor; LI, lateral incisor; Mand, mandibular; Max, maxillary; Mo, molar; PM, premolar. Light stripes indicate the missing tooth rate of maxillary dentition. Dark stripes indicate the missing tooth rate of mandibular dentition

We reviewed five published studies and summarized data from 23 *EDAR*‐related NSTA patients with a total of 13 *EDAR* mutations(Arte et al., 2013; Jonsson et al., 2018; Mumtaz et al., 2020; Zeng et al., 2017; Zhang et al., 2020). The mutation sites of the *EDAR* gene, protein changes, and the types of mutations are given in Table 1. The details of the missing teeth are listed in Table 2. In NSTA patients with *EDAR* mutations, the maxillary lateral incisor had the highest missing rate (69.6%), followed by the mandibular lateral incisor (47.8%), the mandibular second premolars (45.7%), and the maxillary second premolars (41.3%), although this list does not include the third molars. The missing rate of the maxillary central incisors was the lowest (10.9%), followed by the mandibular first molars (13.0%), and the mandibular canines (15.2%). In all, the rate of loss of the molars was lower than that of the anterior teeth (Figure 5).

**TABLE 1:** Summary of *EDAR* ^a^ mutations in patients with non‐syndromic tooth agenesis (NSTA)

Nucleotide change	Amino acid change	Mutant type	Exon	Ref
c.338 G>A	p.Cys113Tyr	Missense	4	—
c.73C>T	p.Arg25*	Nonsense	3	Zeng et al. (2017)
c.973C>T	p.Arg325Trp	Missense	11	Arte et al. (2013)
c.1073G>A	p.Arg358Gln	Missense	12	
c.1135G>A	p.Glu379Lys	Missense	12	
c.1172T>A	p.Met391Lys	Missense	12	
c.1258C>T	p.Arg420Trp	Missense	12	Jonsson et al. (2018)
c.1302G>A	p.Trp434*	Nonsense	12	Mumtaz et al. (2020)
c.404G>A	p.Cys135Tyr	Missense	5	Zhang et al. (2020)
c.1072C>T	p.Arg358*	Nonsense	12	
c.43G>A	p.Val15Ile	Missense	2	
c.871G>A	p.Aal291Thr	Missense	10	
c.1109T>C	p.Val370Ala	Missense	12	

Abbreviation: Ref: reference.

^a^ The reference sequence for *EDAR* is NM_022336.4.

**TABLE 2:** Phenotypes of NSTA patients with *EDAR* mutations

Mutation	Ref	MT^#^		Right							Left						
				7	6	5	4	3	2	1	1	2	3	4	5	6	7
c.73C>T	Zeng et al. (2017)	6/28	Max			*						*			*		
			Mand							*	*				*		
c.338G>A^1^	—	18/28	Max	*	*	*			*	*	*		*		*	*	*
			Mand	*	*	*				*	*	*			*		*
c.338G>A^2^	—	17/28	Max	*	*			*		*	*			*	*	*	*
			Mand	*	*	*				*				*	*	*	*
c.973C>T	Arte et al. (2013)	2/28	Max						*			*					
			Mand														
c.973C>T	Arte et al. (2013)	4/28	Max									*					
			Mand						*	*	*						
c.973C>T	Arte et al. (2013)	9/28	Max			*		*	*			*	*		*		
			Mand						*			*			*		
c.1073G>A	Arte et al. (2013)	5/28	Max						*			*					
			Mand							*	*	*					
c.1135G>A	Arte et al. (2013)	4/28	Max						*			*					
			Mand						*			*					
c.1135G>A	Arte et al. (2013)	3/28	Max						*			*					
			Mand			*											
c.1135G>A	Arte et al. (2013)	10/28	Max			*	*	*	*			*	*		*		
			Mand				*							*	*		
c.1172T>A	Arte et al. (2013)	6/28	Max						*			*					
			Mand			*			*			*			*		
c.1302G>A	Mumtaz et al. (2020)	4/28	Max					*	*	*		*					
			Mand														
c.1302G>A	Mumtaz et al. (2020)	8/28	Max						*			*					
			Mand						*	*		*	*	*		*	
c.1302G>A	Mumtaz et al. (2020)	5/28	Max			*			*			*					
			Mand							*	*						
c.1302G>A	Mumtaz et al. (2020)	6/28	Max						*			*					
			Mand					*	*			*	*				
c.404G>A	Zhang et al. (2020)	12/28	Max	*		*	*	*				*	*	*	*		*
			Mand			*								*	*		
c.1072C>T	Zhang et al. (2020)	9/28	Max				*		*			*					
			Mand					*	*	*	*	*	*				
c.319A>G	Zhang et al. (2020	8/28	Max			*	*	*					*	*	*		
			Mand			*									*		
c.319A>G	Zhang et al. (2020)	15/28	Max		*		*		*			*		*		*	
			Mand		*		*		*	*	*	*		*	*	*	
c.319A>G	Zhang et al. (2020)	8/28	Max	*		*	*							*	*		
			Mand			*			*						*		
c.871G>A	Zhang et al. (2020)	22/28	Max	*		*	*	*	*			*	*	*	*		*
			Mand	*		*	*	*	*	*	*	*	*	*	*		*
c.1138A>C	Zhang et al. (2020)	6/28	Max											*	*		
			Mand	*		*									*		*
c.1138A>C	Zhang et al. (2020)	6/28	Max		*			*					*			*	
			Mand						*			*					

Abbreviations: *Indicates missing teeth; ^1^, Phenotype of proband 4; ^2^, Phenotype of proband 4’s mother, Family 4; Mand, mandibular; Max, maxillary; MT^#^, number of missing permanent teeth, excluding the third molars; Ref, reference.

### 3.5 Phenotypes of NSTA patients with *EDA* mutations at codon 289

**FIGURE 6:**
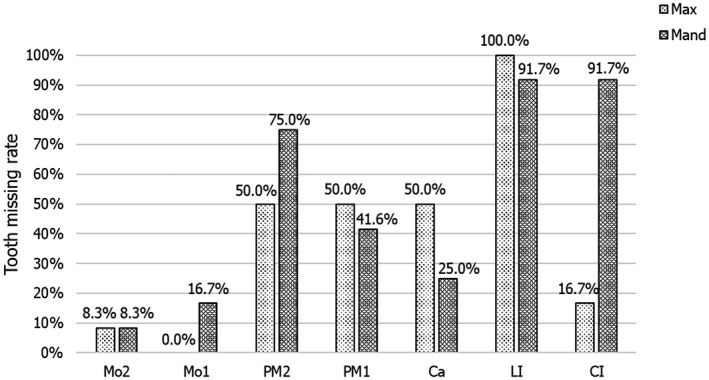
Tooth missing rate of NSTA patients with *EDA* mutations at codon 289 (excluding the third molars). Ca, canine; CI, central incisor; LI, lateral incisor; Mand, mandibular; Max, maxillary; Mo, molar; PM, premolar. Light stripes indicate the missing tooth rate of maxillary dentition. Dark stripes indicate the missing tooth rate of mandibular dentition

Two mutations (c.865C>T, p.Arg289Cys and c.866G>A, p.Arg289His) at codon 289 were identified in our study as well as in previous studies. Another missense amino acid change (c.866G>T, p.Arg289Leu) occurring at the same position was reported by Lee et al. (Table 3; Lee et al., 2014; Ruiz–Heiland et al., 2016; Song et al., 2009)). The p.Arg289Leu and p.Arg289Cys mutations caused a change from a positively charged residue to a non‐polar residue. Although the p.Arg289His mutation did not cause a change in physicochemical properties, the volume of the side‐chain decreased. Phenotype analysis shows that the reported mutations at codon 289 of *EDA* were more likely to affect the maxillary and mandibular lateral incisors (100%, 91.7%), the mandibular central incisors (91.7%), and the mandibular second premolars (75.0%). However, the maxillary and mandibular second molars (8.3%, 8.3%), mandibular first molars (16.7%), and maxillary central incisors (16.7%) were less affected. Interestingly, maxillary first molars are present in all patients (Figure 6).

**TABLE 3:** Phenotypes of NSTA patients with *EDA* ^a^ mutations at codon 289

Mutation	Amino acid change	Ref	MT^#^		Right							Left						
					7	6	5	4	3	2	1	1	2	3	4	5	6	7
c.865C>T^1^	p.Arg289Cys	—	8/28	Max						*			*					
				Mand			*			*	*	*	*			*		
c.865C>T	p.Arg289Cys	Song et al. (2009)	15/28	Max			*	*	*	*			*	*	*	*		
				Mand			*	*	*	*	*				*	*		
c.866G>A^2^	p.Arg289His	—	10/28	Max			*			*			*			*		
				Mand			*			*	*	*	*			*		
c.866G>A	p.Arg289His	Ruiz‐Heiland et al. (2016)	6/28	Max						*			*					
				Mand						*	*	*	*					
c.866G>A	p.Arg289His		12/28	Max				*	*	*			*	*	*			
				Mand				*		*	*	*	*			*		
c.866G>T	p.Arg289Leu	Lee et al. (2014)	24/28	Max	*		*	*	*	*	*	*	*	*	*	*		
				Mand		*	*	*	*	*	*	*	*	*	*	*	*	*

Abbreviations: *indicate missing teeth; ^1^, Phenotype of proband 1; ^2^, Phenotype of proband 2; Max, maxillary; Mand, mandibular; MT^#^, number of missing permanent teeth, excluding the third molars; Ref, reference.

^a^ The reference sequence for *EDA* is NM_001399.5.

## 4 DISCUSSION

Mutations of the *EDAR* gene can result in HED (Chaudhary et al., 2017; Feng et al., 2018). The Human Gene Mutation Database (HGMD Professional, 2018.3) has 62 registered pathogenic variants of *EDAR*, of which 50 have the HED phenotype(Parveen et al., 2019). In recent years, several *EDAR* mutations in NSTA patients have been identified (Arte et al., 2013; Jonsson et al., 2018; Mumtaz et al., 2020; Zeng et al., 2017; Zhang et al., 2020). In our study, a new *EDAR* heterozygous nucleotide substitution c.338G>A (p.(Cys113Tyr); NM_022336.4) (frequency <0.005 in all populations reported in the gnomAD database) was detected in proband 4 by whole‐exome sequencing. This mutation was not included in the Exome Variant Server. The affected individuals in family 4, proband 4, and his mother were identified as heterozygous for the substitution, while the unaffected individuals, his father, and brother, were wild‐type at this position, indicating that the variant cosegregated with the disease in this family. According to SIFT, Polyphen2, and MutationTaster the effects of the variant were predicted to be deleterious (0.00), probably damaging (0.996), and disease‐causing (1.00), respectively. A cross‐species alignment of EDAR amino acid sequences showed that Cys113 was conserved across seven species. It is likely that this variant is the underlying cause of the phenotype in family 4.

EDAR is a type I transmembrane protein and a member of the TNF receptor superfamily. It has a cysteine‐rich domain in the extracellular region (LBD) (encoded by exons 2, 3, 4, and 5), as well as a death domain in its intracellular region (encoded by exon 12). EDAR interacts with extracellular EDA and intracellular EDARADD via the extra‐ and intracellular regions to form a complex. This, in turn, activates downstream NF‐κB to mediate the transcription of the target gene(Kumar et al., 2001; Masui et al., 2011; Okita et al., 2019; Outi et al., 2001; Parveen et al., 2019). The c.338G>A p.(Cys113Tyr) mutation in exon 4 occurs in the LBD that binds to EDA. Structural modeling of the mutant LBD of EDAR protein showed that the p.Cys113Tyr mutation resulted in the substitution of the hydrophobic residue Cys113 by a polar amino acid Tyr with an aromatic ring, and a longer side‐chain compared with Cys. The size of the mutant amino acid Tyr is remarkably different from that of the wild‐type amino acid Cys and may result in the conformational change (Figure 4). We speculated that the p.Cys113Tyr substitution of EDAR might reduce its affinity with EDA, and subsequently affect their interaction, ultimately affecting the activation of downstream NF‐κB signaling and resulting in the failure of tooth formation. Therefore, further functional analysis of *EDAR* mutations is needed to clarify the pathogenic mechanism of non‐syndromic congenital tooth agenesis.

By studying the information of all NSTA patients with *EDAR* mutations, we found that more than half (7/13) of *EDAR* mutations in NSTA patients were concentrated in the death domain encoded by exon 12, and the majority of caustive variants (10/13) were missense mutations (Table 1). We also observed that the most common missing teeth were the maxillary lateral incisors (69.6%), followed by the mandibular lateral incisors (47.8%), the mandibular second premolars (45.7%), and the maxillary second premolars (41.3%). The maxillary central incisors and mandibular first molars were the least likely to be affected (Table 2; Figure 5). The lateral incisors, as well as second premolars, were the most commonly involved, which is consistent with the results of *EDAR*‐associated nonsyndromic tooth agenesis reported by Zhang (L. Zhang et al., 2020). In addition, the number of permanent teeth lost in *EDAR*‐related NSTA patients reported by others ranged from 2 to 22 (Arte et al., 2013; Jonsson et al., 2018; Mumtaz et al., 2020; Zeng et al., 2017; Zhang et al., 2020). In the present study, the proband and his mother had 18 and 17 permanent teeth missing, respectively. In this study, we report the absence of maxillary center incisors in two patients, although the general trend of *EDAR* genotype–phenotype was unchanged.

According to Yu et al. (2019), over the past two decades, 198 different mutations had been detected that are responsible for NSTA, of which 27 are derived from *EDA*. Previous studies revealed a clear link between the genotype and phenotype for congenital tooth deficiency(Han et al., 2008; He et al., 2016; Wong et al., 2018; Zhang et al., 2011). Han et al. (2008) studied 24 NSTA patients with *EDA* mutations and conducted statistical analysis on the number of missing teeth in each position of dentition. They found that the lateral incisors were the most likely to be affected, followed by the mandibular central incisors; and the least likely missing teeth were the maxillary central incisors and first permanent molars. The results also confirmed that these characteristics were specific phenotypes of NSTA caused by *EDA* mutations(He et al., 2016; Zhang et al., 2020).

In our research, three mutations were found in the *EDA* gene of nonsydromic oligodontia Patients 1–3. The probands shared the *EDA* mutations with their mothers (Figure 3), indicating that the mutant alleles were inherited from the maternal line in Families 1, 2, and 3. Two of the mutations (c.865C>T and c.866G>A) observed in this study were located in the TNF domain of *EDA*. The TNF homology domain forms a homotrimer, which is required for interaction with the receptor at the monomer–monomer interface (Hymowitz et al., 2000). Song et al. (2009) carried out homology modeling and 3D structural analysis on the EDA protein and found that in the wild‐type EDA protein structure, the Arg residue at codon 289 is located at the outer surface of the homotrimer and makes structural hydrogen bonds with Asn at codon 272. In addition, Arg at codon 289 forms hydrogen bonds and electrostatic interactions with Glu at codon 308 of the adjacent homotrimer(s) to stabilize the multi‐trimer asymmetric unit (Lee et al., 2014; Ruiz–Heiland et al., 2016; Song et al., 2009). Variants at the p.Arg289 location would abolish these interactions and reduce protein stability. Therefore, codon 289 mutations in the TNF domain partly impact on the binding of EDA protein to EDA receptor.

However, it is worth noting that although all changes occurred at p.Arg289 of EDA, the phenotypes of patients were slightly different. We performed a genotype–phenotype analysis and found that all maxillary lateral incisors are affected (100%), followed by mandibular incisors (91.7%) and mandibular second premolars (75.0%). In contrast, the second molars (8.3%), mandibular first molars(16.7%), and maxillary central incisors (16.7%) were less affected. It is particularly interesting that the maxillary first molars were present in all patients (0.0%) (Figure 6). Our phenotypic analysis of the mutations at codon 289 of *EDA* is consistent with the typical phenotype resulting from *EDA* gene mutations (Zhang et al., 2020). However, previous reports (Lee et al., 2014; Ruiz–Heiland et al., 2016; Song et al., 2009) indicate that the mutations at codon 289 of *EDA* do not affect the development of maxillary first molars. The mechanism that causes the slight changes in the phenotype of teeth loss due to protein mutations remains to be studied.

Interestingly, Ruiz–Heiland et al. (2016) reported two patients with the c.866G>A (p.Arg289His) mutation in the *EDA* gene who presented with oligodontia and sparse hair, with six and 12 missing permanent teeth, respectively. In contrast, oligodontia (10 missing permanent teeth) was the only clinical manifestation in Patient 2 in our study (Figure 1). Maxillary central incisors, as well as maxillary and mandibular molars, were present in all three patients, while the variant preferentially affected maxillary lateral incisors and mandibular incisors. We reviewed the literature and found that the *EDAR* missense variant, c.1258C>T (p.Arg420Trp) had been reported to be associated with NSTA and HED (Jonsson et al., 2018; Wohlfart & Schneider, 2019). Furthermore, Zeng et al. (2017) identified a *WNT10A* mutation c.742C>T (p.Arg248*) in a patient with NSTA, which also was reported in a patient with STA (HED) (Cluzeau et al., 2011). It was proposed that some cases of STA and NSTA, caused by mutations of the same gene, represent the same disease but with phenotypic variability. It can be speculated that this phenomenon is related to epigenetic regulation or other factors although this hypothesis remains to be investigated.

Mutations in different genes cause characteristic phenotypes. Zhang et al. (2020) compared the phenotypic characteristics of *EDAR*‐ and *EDA*‐related NSTA patients and demonstrated that the mandibular premolars are more sensitive to *EDAR* mutations, while the anterior teeth are more sensitive to *EDA* mutations. Our current study extends the spectrum of mutants related to non‐synchromatic *EDAR*‐associated NSTA and shows a greater tendency for the absence of lateral incisors and second premolars in *EDAR*‐associated NSTA, which is similar to the phenotypic characteristics of *EDA*‐associated NSTA. According to our results, *EDAR*‐ and *EDA*‐related NSTA have similar trends in the location of the affected teeth. Mumtaz et al. (Mumtaz et al., 2020) found that permanent incisors were most commonly involved in *EDAR*‐related tooth agenesis cases, which is consistent with our findings. Further investigations of the spectrum of *EDAR* mutants are required to fully understand the phenotypic characteristics of *EDAR*‐related NSTA.

## 5 CONCLUSION

In this study, we identified a new *EDAR* variant c.338G>A p.(Cys113Tyr) in a Chinese family with NSTA. In addition, we studied previously reported *EDAR* mutations and summarized their genotype–phenotype correlation in NSTA patients. This allowed us to expand the *EDAR* mutation spectrum as well as providing a genetic basis for the pathogenesis of congenital tooth agenesis. This research could help in preconception genetic counseling, prenatal screening, and fetus diagnosis, which contribute to disease status prediction in NSTA families.

## CONFLICT OF INTEREST

The authors declare no conflict of interest.

## AUTHOR CONTRIBUTIONS

W.J.S. conceived and designed the experiments; G.Z.Z. and Q.Q.D. contributed reagents and methodology; J.B.R., W.J.S., S.Y., C.Y.L., Y.H., and Y.L. provided clinical cases; X.T.K. and H.Y.Z. performed the experiments; W.J.S., J.B.R., H.Y.Z., and X.T.K. analyzed the data; H.Y.Z. and X.T.K. wrote the paper; W.J.S. and L.Q.M: revised the manuscript critically. All authors read and approved the final manuscript.

## Data Availability

These sequence data have been submitted to the ClinVar databases [ClinVar (https://www.ncbi.nlm.nih.gov/clinvar)] under Submission ID: SUB8384712; Accession: VCV000986783.1; and Variation ID: 986783. Other data that support the findings of this study are available from the corresponding author upon reasonable request.
